# Prediction of protein structural classes by different feature expressions based on 2-D wavelet denoising and fusion

**DOI:** 10.1186/s12859-019-3276-5

**Published:** 2019-12-24

**Authors:** Shunfang Wang, Xiaoheng Wang

**Affiliations:** Department of Computer Science and Engineering, School of Information Science and Engineering, Yunnan University, Kunming, 650504 People’s Republic of China

**Keywords:** Prediction of protein structural classes, Different feature expressions, Parallel 2-D wavelet denoising, Fusion

## Abstract

**Background:**

Protein structural class predicting is a heavily researched subject in bioinformatics that plays a vital role in protein functional analysis, protein folding recognition, rational drug design and other related fields. However, when traditional feature expression methods are adopted, the features usually contain considerable redundant information, which leads to a very low recognition rate of protein structural classes.

**Results:**

We constructed a prediction model based on wavelet denoising using different feature expression methods. A new fusion idea, first fuse and then denoise, is proposed in this article. Two types of pseudo amino acid compositions are utilized to distill feature vectors. Then, a two-dimensional (2-D) wavelet denoising algorithm is used to remove the redundant information from two extracted feature vectors. The two feature vectors based on parallel 2-D wavelet denoising are fused, which is known as PWD-FU-PseAAC. The related source codes are available at https://github.com/Xiaoheng-Wang12/Wang-xiaoheng/tree/master.

**Conclusions:**

Experimental verification of three low-similarity datasets suggests that the proposed model achieves notably good results as regarding the prediction of protein structural classes.

## Background

Protein structural class prediction is a heavily researched subject in bioinformatics and performs a vital role in many related fields and applications, such as protein functional analysis, protein folding recognition, protein binding, rational drug design and so on [1–11]. However, in the light of newly discovered proteins, it will take time and money to determine the structure of proteins by traditional experimental methods, so many computational methods have been proposed to predict protein structural classes. Because the sequence of amino acids determines the specific spatial structure of protein, the method of predicting structural classes by sequence is a concise and effective way, which can help guide the direction of biological experiment, save the cost of biological experiment and provide useful information for a heuristic approach [9–12]. In particular, when the feature information of proteins is extracted, they often contain considerable redundant information, resulting in an unsatisfactory recognition rate for structural classes of protein.

To solve the problems of redundant information and low recognition rates, many computational methods have been proposed to predict protein structural classes during the past 30 years. One such method is the feature extraction method based on the information in amino acid sequences. Initially, amino acid composition [12, 13] (AAC) was used to extract the feature information. This method calculated the proportion of twenty amino acid residues in the sequence and expressed the feature information of the sequence by numerical vectors. Pseudo amino acid composition [14–19] (PseACC) was also used to extract its feature information. This method considered not only the composition of amino acid residues but also their hydrophobicity and other physical and chemical properties. In addition, peptide composition [20, 21] was adopted to extract its feature information. Compared with the previous two methods, this method considered the sequence factor between amino acid residues. These methods have achieved good prediction results on high similarity datasets but poor results on low similarity datasets. The prediction accuracy of these methods can reach more than 90% on high similarity datasets but only approximately 50% on low similarity datasets. Some improved feature extraction methods have been proposed. Lukasz et al. proposed the SCPRED method [22], which constructed feature vectors based on predictive secondary structure. Zhang proposed a TPM matrix to represent the feature on the predictive secondary structure [23], and Dai et al. [24] proposed a statistical feature method on the predictive secondary structure feature, which takes the secondary structure feature as part of the feature vector. In Ding [25], a multidimensional representation vector is constructed to predict protein secondary structural classes. Some methods for fusing multiple features such as feature selection [26] are also proposed. Chen et al. proposed the fusion of multiple features [27], which combined the derived structure information of sequences with the physicochemical properties [28]. Nanni et al. proposed a new feature fusion method based on the features of the primary sequence and the features of the secondary structure based on prediction [29]. Wang et al. [30] fused the improved simplified PSSM with secondary structure features. In addition, some other classical feature extraction methods have been proposed, such as Dehzangi et al., who used piecewise distribution and piecewise autocovariance ideas [31]. It is noted that it is hard for the above feature fusion algorithms to reduce the redundancy of feature information, which thus makes prediction accuracy hard to improve. Based on this properity, Liu et al. used a recursive feature selection algorithm to select the optimal feature vector [32].

The second is the classification algorithm. As far as the four common cases of structural classes, all-α, all-β, α/β and α + β are concerned, how to distinguish them accurately is essential an efficient multi-classification problems. Multiple classification and various machine learning algorithms have been applied to protein classification prediction, such as neural networks, fuzzy clustering, Naive Bayes, support vector machines (SVM), K-nearest neighbors (KNN) and the correlation coefficients methods [12, 33–40]. However, because the dataset used in protein structure prediction is usually small sample data, and the neural network classification algorithm requires a large amount of data, its performance cannot be fully developed. The fuzzy clustering algorithm also faces the same problem because the sample size is too small to cluster well, resulting in poor prediction results. For Naive Bayesian classification, the premise is that there is no correlation between the features and attributes, and it is sensitive to the form of data input. These factors affect the performance of classification prediction to a certain extent. Support Vector Machine can also play a role in classification performance when there are few data samples, but the process of searching parameters is highly time-consuming. The K-nearest neighbor algorithm is simple in theory, easy to implement, simple and efficient. This algorithm is also suitable for classification of small sample data. Later, some improved classification algorithms have been proposed. For example, Chen et al. proposed a method of fusing multiple support vector machines [41]. This method divides the extracted feature vectors into three parts, each part is input into a corresponding classifier, and then synthesizes the classification results of the three parts, voting to determine the category of the samples to be tested. The improved method is to fuse the same classifier. After that step, the fusions of different types of classifiers have been proposed, such as Dehzangi and other classifiers [42]. The classifiers are AdaBoost, M1, LogitBoost, SVM, MLP and Naive Bayes. However, the problem that redundant information in the feature vector affects the generalization ability of the model has not been solved by these methods.

In this article, to deal with this problem, the newly developed model for predicting structural classes of proteins is put forward based on different feature expression methods, known as PWD-FU-PseAAC. In order to prove the superiority of the proposed method, the extracted feature vectors are based on the primary sequence information of proteins. First, the features of the primary sequence of proteins are distilled by the traditional feature expression methods, type 1 pseudo amino acid composition (PseAAC) [43] and type 2 pseudo amino acid composition [44]. Since type 1 PseAAC is popularly used in many researches, here we explain a little about type 2 PseAAC. In Chou [44], type 2 PseAAC is also called ‘amphiphilic pseudo amino acid composition’, whose form is like AAC except much more information about the distribution of the hydrophobic and hydrophilic amino acids of a protein. Second, two-dimensional multiscale wavelet denoising is used to process the feature vectors extracted by two feature expression methods, removing the redundant information from them. In the field of mathematics, a new direction of rapid and groundbreaking development is wavelet analysis, which has been increasingly widely utilized in the field of bioinformatics, particularly for protein structural prediction and functional analysis. This analysis has the characteristics of local transformation in the time domain and frequency domain and may efficaciously extract information from signals and perform multiscale fine analysis of functions or signals through scaling and translation operations. Wavelet denoising [45] is one of the significant branches of wavelet analysis, which can efficaciously eliminate redundant information of the extracted feature vectors, making the information more stable and efficacious, and improving the accuracy of prediction. Due to the complexity of the protein structure, it can be reasonably to employ two-dimensional (2-D) wavelet de-noising rather than one-dimensional (1-D) wavelet de-noising. To illustrate the validity of 2-D wavelet denoising, it is compared with the 1-D wavelet denoising in the following experimental parts. Third, the new feature vectors are obtained by fusing the two different feature vectors after denoising. Finally, the optimal feature vectors are treated as input data of the KNN to predict structural classes of proteins. To estimate the performance of our presented model, we adopt the jackknife test as a validation method to carry out relevant experimental analysis on the three low-similarity datasets. The final experimental outcomes indicate that our model has higher overall prediction accuracies than other methods.

## Methods

### Datasets

To compare with current methods fairly and objectively, three low-similarity benchmark datasets, the 25PDB [46], the 1189PDB [47] and the 640PDB [48], are selected as our experimental datasets, which are structural protein sequences with internal similarities of less than 25, 40 and 25%, respectively. The datasets have four categories, the details of which are shown in Table 1.

**Table 1:** Detailed information of the two datasets

Dataset	Number of proteins				
	all-α	all-β	α/β	α + β	Total
25PDB	443	443	346	441	1673
1189PDB	223	294	334	241	1092
640PDB	138	154	177	171	640

### Feature extraction

In this article, the traditional feature expression methods, two types of pseudo amino acid compositions, are applied to convert the primary sequences of protein into numerical feature vectors. As known to all, pseudo amino acid composition is an improved expression on the basis of amino acid composition, not only considering the frequency of amino acid residues in the sequence but also considering the physicochemical properties of amino acid residues. There are two types of pseudo amino acid composition: parallel correlation type and sequence correlation type. For convenience, the pseudo amino acid composition of the parallel correlation type is called type 1 pseudo amino acid composition, and that of the sequence correlation type is called type 2 pseudo amino acid composition.

1. Type 1 pseudo amino acid composition

Type 1 pseudo amino acid composition was proposed by Chou in 2001 [43]. This composition considers not only the hydrophilicity and hydrophobicity of amino acid residues, but also the quality of side chain groups of amino acid residues. Type 1 pseudo amino acid composition is used to extract the features of structural protein sequences.

Thus, a protein sequence can be transformed into 20+ *λ* dimensional numerical vectors, that is, *P*_*PseAAC* _ *type*1_ = [*p*_1_, *p*_2_, ......, *p*_20 + *λ*_]^*T*^, where *p*_*u*_ can be calculated from eq. (1):

1 $$ {p}_u=\left\{{\displaystyle \begin{array}{l}\frac{f_u}{\sum \limits_{i=1}^{20}{f}_i+\omega \sum \limits_{j=1}^{\lambda }{\theta}_j}\left(1\le u\le 20\right)\\ {}\frac{\omega {\theta}_{u-20}}{\sum \limits_{i=1}^{20}{f}_i+\omega \sum \limits_{j=1}^{\lambda }{\theta}_j}\left(20+1\le u\le 20+\lambda \right)\end{array}}\right. $$

where *f*_*i*_ is the frequency of 20 amino acid residues in protein sequence P; *w* is the weight factor, which is generally set to 0.05; *λ* is the hierarchical factor, which is less than the total length of the sequence *N*; *θ*_*j*_ is the sequence correlation coefficient of the j-th layer, which can be calculated from eq. (2):

2 $$ {\theta}_{\lambda }=\frac{1}{L-\lambda}\sum \limits_{i-1}^{L-\lambda}\varPhi \left({R}_i,{R}_{i+\lambda}\right) $$

In addition:

3 $$ \varPhi \left({R}_i,{R}_j\right)=\frac{1}{3}\left\{{\left[{H}_1\left({R}_j\right)-{H}_1\left({R}_i\right)\right]}^2+{\left[{H}_2\left({R}_j\right)-{H}_2\left({R}_i\right)\right]}^2+{\left[{H}_3\left({R}_j\right)-{H}_3\left({R}_i\right)\right]}^2\right\} $$

Among them, *H*_1_(*R*_*i*_), *H*_2_(*R*_*i*_) and *H*_3_(*R*_*i*_) represent the hydrophobicity, hydrophilicity and the quality of side chain groups of amino acid residues, respectively.

(2) Type 2 pseudo amino acid composition

Type 2 pseudo amino acid composition was proposed by Chou in 2005 [44] because it considers the hydrophilicity and hydrophobicity of amino acid residues, also known as amphipathic pseudo amino acid composition. In this article, type 2 pseudo amino acid composition is also used to extract the features of structural protein sequences.

Thus, a protein sequence can be transformed into 20+ 2*r* dimensional numerical vectors, with *P*_*PseAAC* _ *type*1_ = [*p*_1_, *p*_2_, ......, *p*_20 + 2*r*_]^*T*^, where *p*_*u*_ can be calculated from equation (4):

4 $$ {p}_u=\left\{{\displaystyle \begin{array}{l}\frac{f_u}{\sum \limits_{i=1}^{20}{f}_i+\omega \sum \limits_{j=1}^{2r}{\tau}_j}\left(1\le u\le 20\right)\\ {}\frac{\omega {\tau}_u}{\sum \limits_{i=1}^{20}{f}_i+\omega \sum \limits_{j=1}^{2r}{\tau}_j}\left(20+1\le u\le 20+2r\right)\end{array}}\right. $$

where *r* is the hierarchical factor, which is less than the total length of the sequence *N*; *τ*_*j*_ is the sequence correlation coefficient of the j-th layer, which can be calculated from eq. (5):

5 $$ \left\{{\displaystyle \begin{array}{l}{\tau}_1=\frac{1}{L-1}\sum \limits_{i=1}^{L-1}{H}_{i,i+1}^1\\ {}{\tau}_2=\frac{1}{L-1}\sum \limits_{i=1}^{L-1}{H}_{i,i+1}^2\\ {}{\tau}_3=\frac{1}{L-2}\sum \limits_{i=1}^{L-2}{H}_{i,i+2}^1\\ {}{\tau}_4=\frac{1}{L-2}\sum \limits_{i=1}^{L-2}{H}_{i,i+2}^2\\ {}\cdots \cdots \cdots \\ {}{\tau}_{2\lambda -1}=\frac{1}{L-\lambda}\sum \limits_{i=1}^{L-\lambda }{H}_{i,i+\lambda}^1\\ {}{\tau}_{2\lambda }=\frac{1}{L-\lambda}\sum \limits_{i=1}^{L-\lambda }{H}_{i,i+\lambda}^2\end{array}},,,\left(\lambda <L\right)\right. $$

In addition:

6 $$ \Big\{{\displaystyle \begin{array}{l}{H}_{i,j}^1={H}^1\left({R}_i\right)\ast {H}^2\left({R}_j\right)\\ {}{H}_{i,j}^2={H}^2\left({R}_i\right)\ast {H}^2\left({R}_j\right)\end{array}} $$

where *H*^1^(*R*_*i*_) refer to the hydrophobicity of amino acid residues, and *H*^2^(*R*_*i*_) refer to the hydrophilicity of amino acid residues.

### Two-dimensional wavelet denoising

The process of wavelet denoising includes the following three parts: wavelet transform, processing of wavelet coefficients and wavelet inverse transform [49]. There are three commonly used methods of wavelet denoising: wavelet threshold denoising, modulus maximum denoising and spatial correlation denoising. To suppress the noise in the high frequency section and remove redundant information, the wavelet threshold denoising method is adopted. In other words, the wavelet denoising method used refers to the wavelet threshold denoising method in this paper.

This method’s decomposition and reconstruction can be expressed as follows:

7 $$ {f}^0\leftrightarrow \left\{{\displaystyle \begin{array}{l}{f}_L^1\leftrightarrow \Big\{\begin{array}{l}{f}_L^2\leftrightarrow \left\{...{f}_L^{n-1}\leftrightarrow \right\{\begin{array}{l}{f}_L^n\\ {}{f}_H^n\Big\{\begin{array}{l}{f}_{HH}^n\\ {}{f}_{HV}^n\\ {}{f}_{HD}^n\end{array}\end{array}\\ {}{f}_H^2\Big\{\begin{array}{l}{f}_{HH}^2\\ {}{f}_{HV}^2\\ {}{f}_{HD}^2\end{array}\end{array}\\ {}{f}_H^1\Big\{\begin{array}{l}{f}_{HH}^1\\ {}{f}_{HV}^1\\ {}{f}_{HD}^1\end{array}\end{array}}\right. $$

where *f*^0^ represents the original signal; ${f}_L^i$ represents the i-th layer low frequency component obtained by wavelet decomposition; ${f}_H^i$ represents the i-th layer high frequency component obtained by wavelet decomposition; It contains three high-frequency components, in which ${f}_{HH}^i$ refers to the horizontal component, ${f}_{HV}^i$ refers to the vertical component and ${f}_{HD}^i$ refers to the diagonal component.

Then, the above can be expressed as:

8 $$ \Big\{{\displaystyle \begin{array}{l}{f}^0={f}_L^0\\ {}{f}_L^{k-1}=\left(\left({f}_{HH}^k\oplus {f}_{HV}^k\oplus {f}_{HD}^k\right)\oplus \left(\left({f}_{HH}^{k+1}\oplus {f}_{HV}^{k+1}\oplus {f}_{HD}^{k+1}\right)\oplus {f}_L^{k+1}\right)\right)\end{array}}\mathrm{k}=1,\mathrm{2...}\mathrm{n} $$

where ⊕ represents the direct orthogonal sum.

In addition, formula (8) can also be expressed as (9):

9 $$ {f}^0={f}_L^n\oplus {\sum}_{k=1}^n\left({f}_{HH}^k\oplus {f}_{HV}^k\oplus {f}_{HD}^k\right) $$

**Fig. 1:**
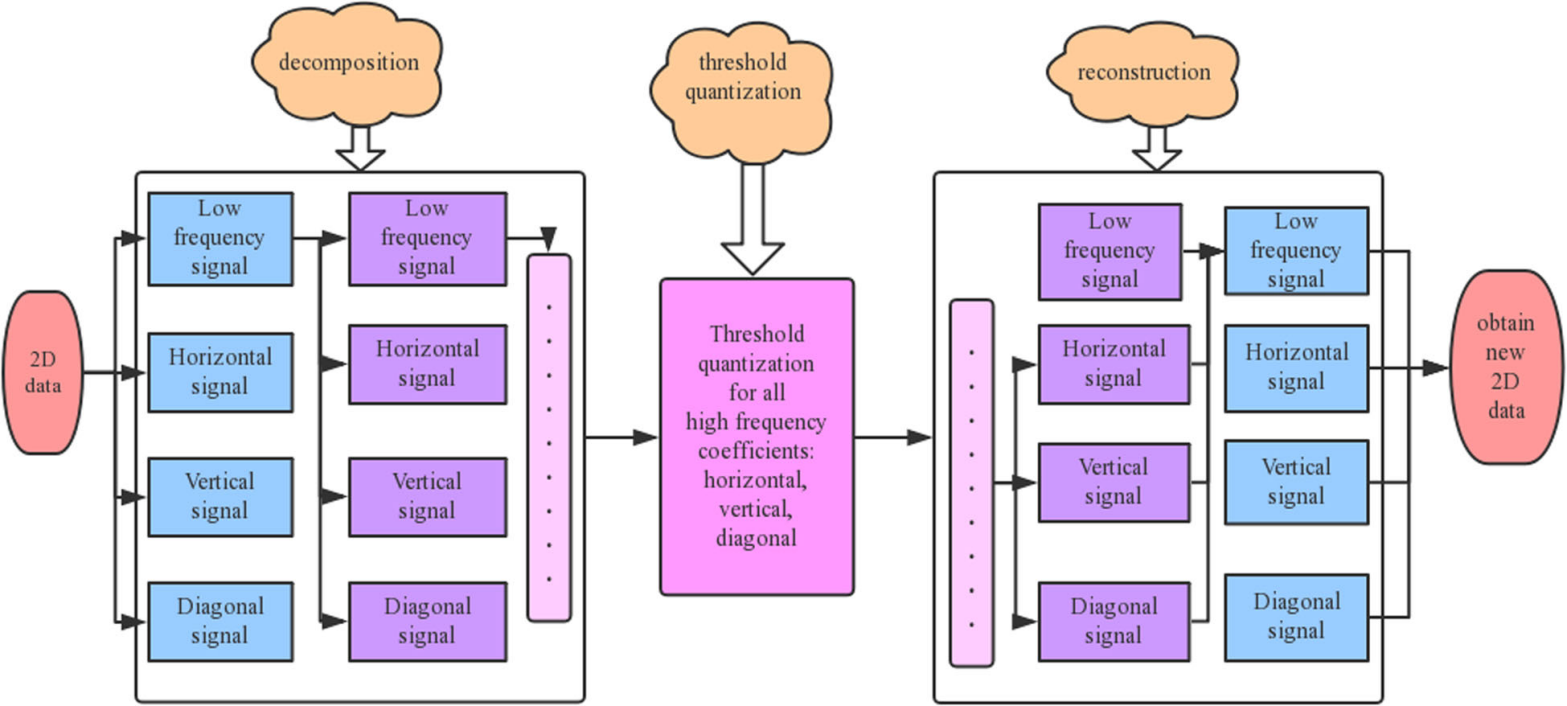
Flow chart of 2-D wavelet denoising

The flow chart of 2-D wavelet denoising is shown in Fig. 1.

In Fig. 1, the input is the original 2-D data and the output is the new obtained 2-D data, the intermediate procedures of the 2-D wavelet denoising is mainly as follows, which is summarized and deduced from references [48–53]:

1) Set the wavelet basis function x, decomposition scale n and threshold value t.

2) Through the wavelet transform, 2-D data are decomposed into four components, one of which is a low frequency component, and the other three of which are high frequency components: a horizontal component, a vertical component and a diagonal component.

3) The low frequency component obtained from step 2 can be further decomposed into a new low frequency component and three new high frequency components: horizontal component, vertical component and diagonal component. Repeat this process until the decomposition scale n is reached.

4) A threshold value is applied to quantize high frequency coefficients obtained by each decomposition.

5) The lastly decomposed and quantized high-frequency component is reconstructed by wavelet transform with the only low-frequency component to form a new low-frequency component. The process is repeated n times upward until the new 2-D data are synthesized.

The algorithm’s pseudocode is shown in Table 2.

**Table 2:** Pseudocode of the 2-D wavelet denoising algorithm

Input: 2-D data, d1 Output: new 2-D data, d2	
1	set x, n, t, j = 0; //set wavelet function, decomposition scale, threshold value and pointer j.
2	(L [j], h1[j], h2[j], h3[j]) = wavedec2(x, d1) //decompose data.
3	(h1[j], h2[j], h3[j]) = threshold(t, h1[j], h2[j], h3[j]); //quantize high frequency coefficients.
4	for→j = 0 to n-1: //the process of decomposition.
5	(L [j + 1], h1[j + 1], h2[j + 1], h3[j + 1]) = wavedec2(x, L [j]);
6	(h1[j + 1], h2[j + 1], h3[j + 1]) = threshold(h, h1[j + 1], h2[j + 1], h3[j + 1]); j = j + 1;
7	for→i = n-1 to 0: //the process of reconstruction.
8	L [i-1] = waverec2(x, L [i], h1[i], h2[i], h3[i]); i = i-1;
9	d2 = waverec2(x, L [i], h1[i], h2[i], h3[i]); //reconstruct data.

Clearly, the key of the wavelet denoising method is undoubtedly to select the value of threshold and threshold function, which has the greatest impact on the effect of wavelet denoising. There are generally three ways to select the value of threshold: default threshold, given threshold and forced threshold. In this article, the default threshold determination model is selected to calculate the value of the threshold because it is convenient and concise. Furthermore, there are two common threshold functions: a soft threshold function and a hard threshold function. We choose a soft threshold function for quantifying because it makes reconstructed signals considerably smoother than the hard one.

### Construction of prediction model

**Fig. 2:**
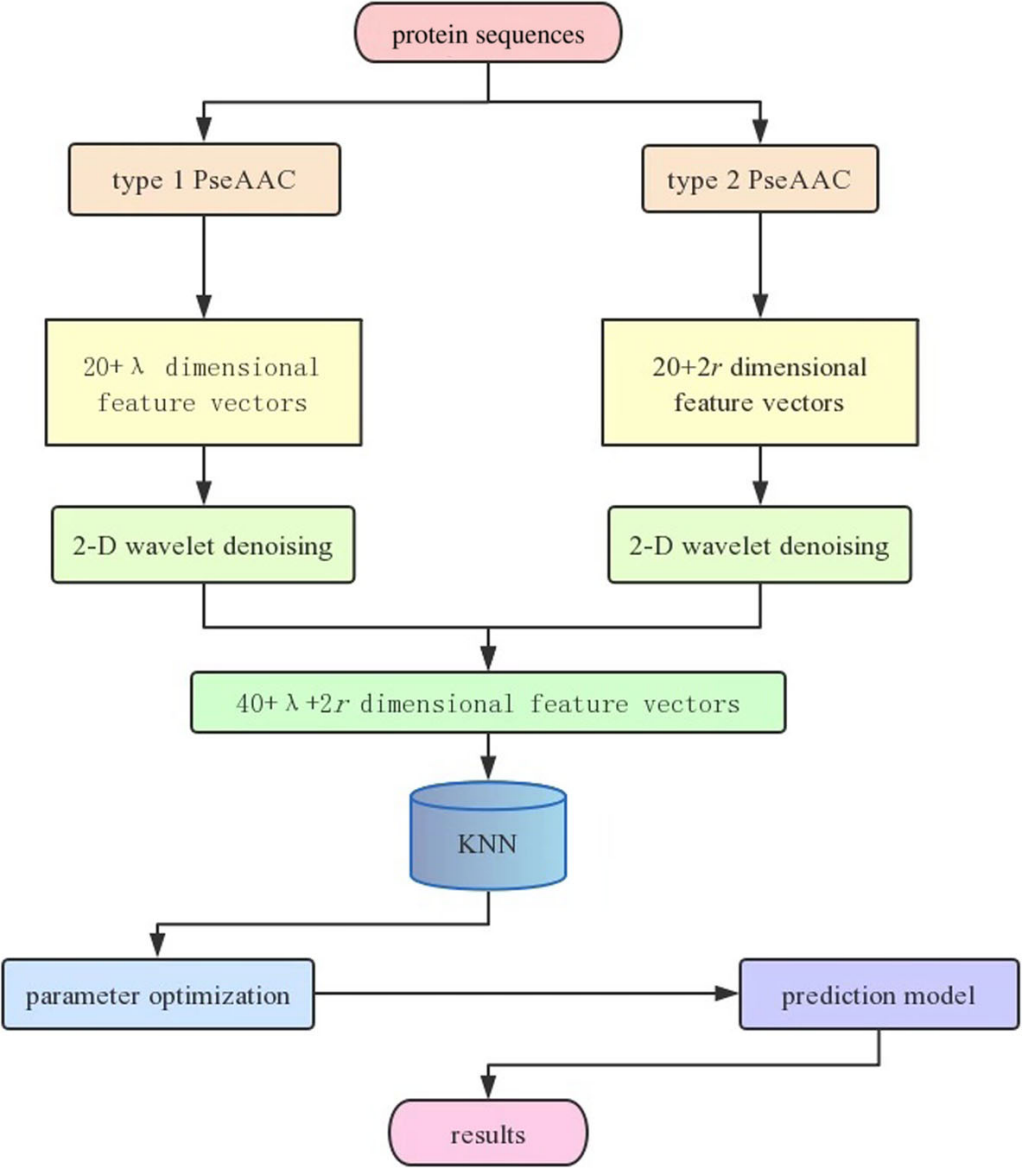
Flow chart of the PWD-FU-PseAAC method

In this article, a new method, called PWD-FU-PseAAC, is proposed to predict the structural classes of protein sequences. First, the feature information of protein sequences is extracted by the traditional feature expression method, type 1 pseudo amino acid composition and type 2 pseudo amino acid composition. Each protein sequence is converted to 20+ *λ* dimensional numerical vectors by type 1 pseudo amino acid composition, and each protein sequence is converted to 20+ 2*r* dimensional numerical vectors by type 2 pseudo amino acid composition. Second two-dimensional wavelet denoising is used to denoise the two feature vectors separately. Then, the two feature vectors after denoising are fused, which refers to splicing the first and last vectors of the two parts to form 40+ *λ* + 2*r* dimensional feature vectors. Moreover, the optimal 40+ *λ* + 2*r* dimensional feature vectors are fed into the KNN classifier for predicting. The jackknife test is used to test the performance of the model on the 25PDB, the 1189PDB and the 640PDB. According to the predicting accuracy, the parameters of the model are adjusted continuously to optimize the performance of the model. Finally, four measures are used to evaluate the performance of the predicting model. The advantages of choosing the classifier KNN are its efficiency and simplicity. Although KNN’s classifying effect is not as good as that of support vector machine (SVM), KNN requires considerably less running time than SVM, as the latter requires considerably effort to determine the optimal parameters. Therefore, considering the classifiers comprehensively, we choose KNN instead of SVM. The flow chart of the model is shown in Fig. 2.

In Fig. 2, new method of PWD-FU-PseAAC is as follows. The feature information of protein sequences is extracted by type 1 pseudo amino acid composition and type 2 pseudo amino acid composition, respectively. Then, 2-D wavelet denoising is used to denoise the two feature vectors, respectively. Next, the two feature vectors after denoising are fused to form a 40+ *λ* + 2*r* dimensional vector, which is entered to the KNN classifier for predicting.

### Performance evaluation

Four validation methods are commonly applied to estimate the performance of the prediction model: the self-consistency test, independent dataset test, k-fold cross-validation and jackknife test [53–57]. Because of the objectivity and strictness of the jackknife test, in this experiment, we make use of it to examine the performance of our prediction model. The sensitivity (Sens), specificity (Spec), overall accuracy (OA) and Matthews correlation coefficient (MCC) are applied to assess the performance of our method. These measures are expressed in the following formula:

10 $$ Sens=\frac{TP}{TP+ FN} $$

11 $$ Spec=\frac{TN}{FP+ TN} $$

12 $$ OA=\frac{TP+ TN}{TP+ TN+ FP+ FN} $$

13 $$ MCC=\frac{TP\times TN- FP\times FN}{\sqrt{\left( TP+ FP\right)\left( TP+ FN\right)\left( TN+ FP\right)\left( TN+ FN\right)}} $$

where *TP* denotes the number of true positives, *FP* denotes the number of false positives, *TN* denotes the number of true negatives, and *FN* denotes the number of false negatives.

## Results and discussion

### Choice of λ and *r* parameters

In this article, two types of pseudo amino acid compositions are used to extract feature vectors, and different parameters of λ and *r* will lead to inconsistency of the feature information contained in the extracted feature vectors, thereby affecting the final prediction results. Therefore, it is necessary to choose the optimal value of λ and *r*, and the range of λ and *r* are 1 to 9, therefore, this section chooses the optimal parameter of λ or *r* between 1 and 9. In this paper, using the 25PDB as the research object, the validity of these feature vectors extracted from two different types of pseudo amino acids is discussed respectively. The wavelet basis function of two-dimensional wavelet denoising is db4, the wavelet decomposition scale is 3, and the K value of the KNN classifier is set to 3. The experimental results of the overall prediction accuracy of protein structural classes and the prediction accuracy of each class are shown in Table 3 and Table 4.

**Table 3:** Prediction results of type 1 PseAAC by different values of λ on the 25PDB

Class	λ								
	Jackknife test(%)								
	1	2	3	4	5	6	7	8	9
all-α	77.43	94.58	88.71	85.10	88.94	88.49	87.36	88.26	87.81
all-β	89.16	90.52	90.52	89.39	88.94	88.04	90.29	90.29	90.52
α/β	78.03	88.73	86.42	83.53	87.57	86.71	86.99	89.31	91.62
α + β	68.03	78.23	76.87	75.28	76.42	75.28	72.11	73.47	71.20
OA	78.18	87.98	85.59	83.32	85.36	84.52	84.04	85.11	84.94

**Table 4:** Prediction results of type 2 PseAAC by different values of *r* on the 25PDB

Class	*r*								
	Jackknife test(%)								
	1	2	3	4	5	6	7	8	9
all-α	76.07	74.49	70.88	73.81	72.23	71.11	71.11	68.17	63.43
all-β	87.81	88.49	85.78	83.75	84.65	83.75	82.39	79.46	79.46
α/β	76.01	79.77	78.90	82.08	85.55	83.82	86.71	85.55	87.57
α + β	61.45	65.76	60.09	62.59	56.46	51.47	50.34	47.62	44.22
OA	75.31	76.99	73.64	75.19	74.12	71.91	71.85	69.34	67.60

From Tables 3 and 4, it can be concluded that different λ_1_ and λ_2_ values do have an impact on the prediction results. When λ and *r* are 2, the overall prediction accuracy is the highest, 87.98 and 76.99% respectively. Therefore, the optimum λ and *r* for both types of pseudo amino acid compositions is 2.

### Choice of the wavelet function and decomposition scale

The traditional feature expression method, type 1 pseudo amino acid composition and type 2 pseudo amino acid composition, are adopted in this article, which still contains considerable redundant information. To obtain more efficacious information, two-dimensional wavelet denoising is used to process the feature vectors extracted by two feature expression methods separately, removing the redundant information from them.

However, the choice of wavelet function and decomposition scale will determine the denoising effect of the models and then further affect the final overall prediction accuracy. To further obtain efficacious information on structural proteins, we chose different wavelet functions and different decomposition scales to examine the effect on the prediction models, including db2, db4, db6, sym2, sym4, sym6, coif1, coif3, bior2.2 and bior2.4, and the decomposition scale from 2 to 5. We discussed the optimal denoising parameters of the feature vectors extracted by type 1 PseAAC and type 2 PseAAC.

**Fig. 3:**
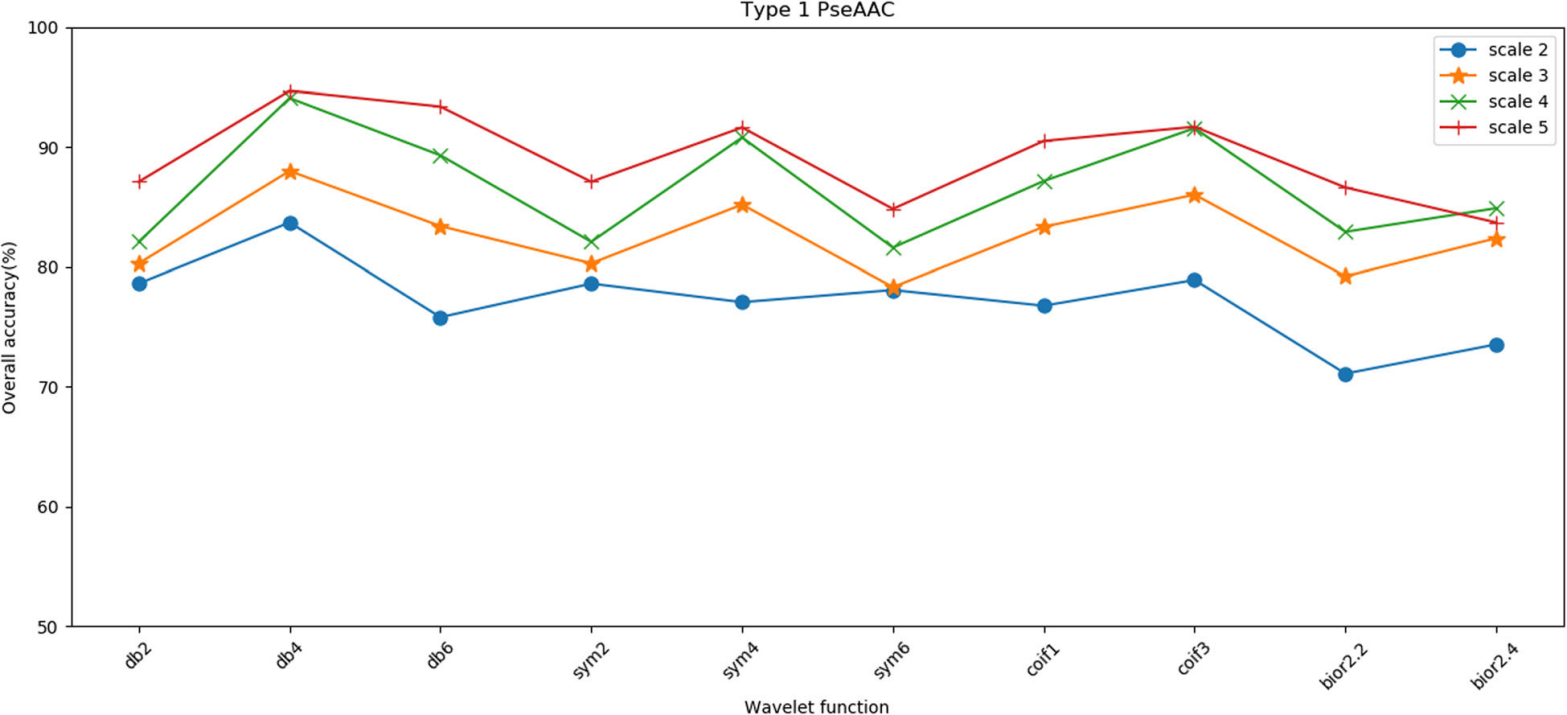
Prediction results by type 1 PseAAC on different decomposition scales and wavelet basis functions on the 25PDB

**Fig. 4:**
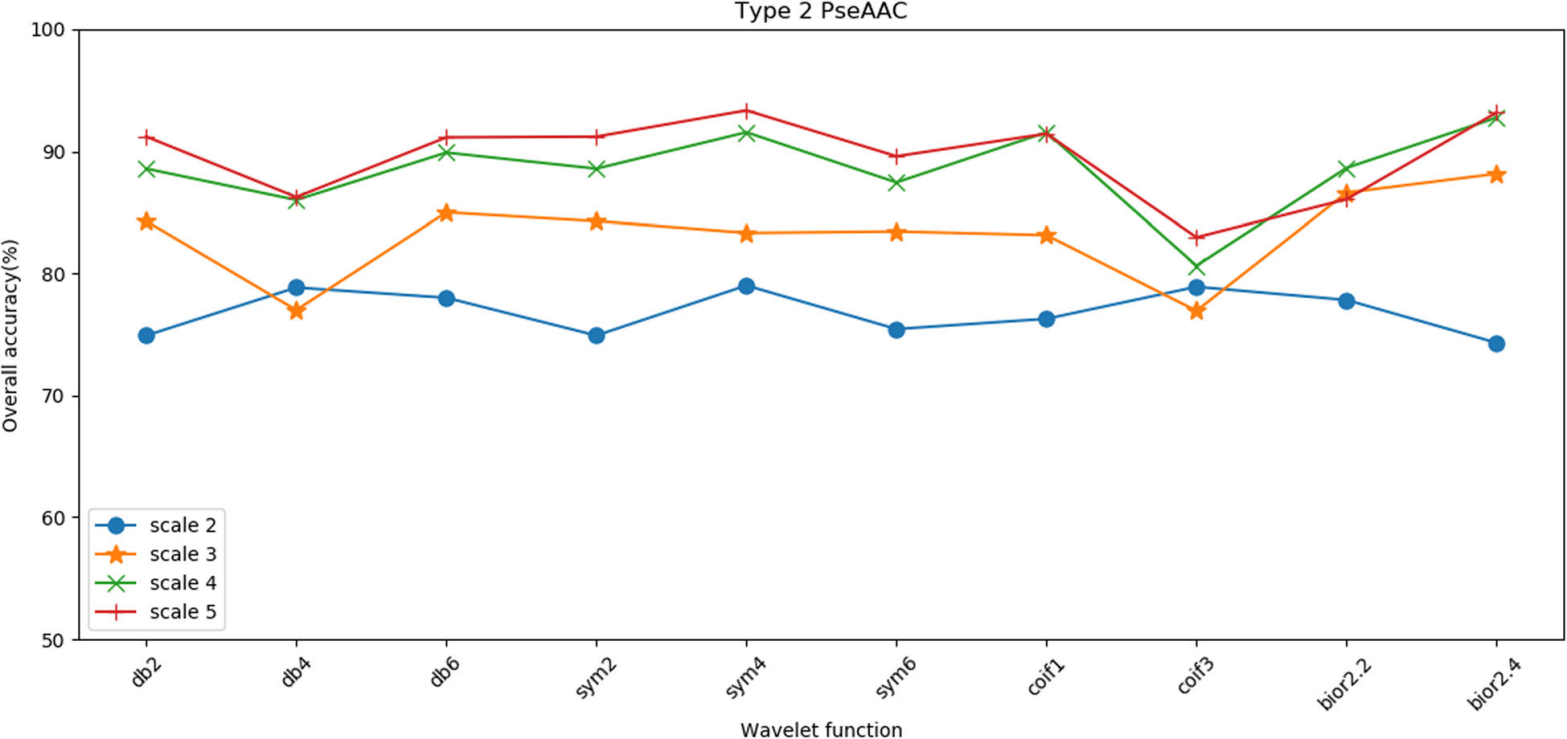
Prediction results by type 2 PseAAC on different decomposition scales and wavelet basis functions on the 25PDB

The 25PDB is selected as the sample for finding the optimal parameters. Table 5 and Table 6 show that the two related factors of the wavelet function and decomposition scale do affect the effect of denoising, thereby affecting the overall prediction accuracy of the method. When the decomposition scale is 5 and the db6 wavelet function is adopted, the effect of wavelet denoising is optimal in Table 5; when the decomposition scale is 5 and the sym4 wavelet function is adopted, the effect of wavelet denoising is optimal in Table 6. Hence, to obtain good prediction results, we choose 5 as the decomposition scale and db4 wavelet as the wavelet function to denoise feature vectors extracted by type 1 pseudo amino acid composition; we choose 5 as the decomposition scale and sym4 wavelet as the wavelet function to denoise feature vectors extracted by type 2 pseudo amino acid composition. In addition, Table 5 and Table 6 show that when the decomposition scale is 2, regardless of the type of wavelet basis function chosen, the overall prediction accuracy is lower than other scales. With the increase of the decomposition scale, the overall prediction accuracy has an upward trend. To describe this trend more intuitively, we drew line charts of the overall prediction accuracy under different wavelet basis functions and decomposition scales, as shown in Figs. 3 and 4.

**Table 5:** Prediction results on the 25PDB by different wavelet functions and different wavelet decomposition scales using type 1 PseAAC

Wavelet	Scales			
	Jackknife test (%)			
	2	3	4	5
db2	78.60	80.27	82.07	87.09
db4	83.68	87.99	94.08	**94.68**
db6	75.79	83.38	89.30	93.37
sym2	78.60	80.27	82.07	87.09
sym4	77.05	85.18	90.79	91.63
sym6	78.06	78.30	81.59	84.82
coif1	76.75	83.32	87.15	90.50
coif3	78.90	86.01	91.57	91.69
bior2.2	71.07	79.20	82.90	86.61
bior2.4	73.52	82.37	84.88	83.68

**Table 6:** Prediction results on the 25PDB by different wavelet functions and different wavelet decomposition scales using type 2 PseAAC

Wavelet	Scales			
	Jackknife test (%)			
	2	3	4	5
db2	74.90	84.28	88.58	91.21
db4	78.84	76.99	86.01	86.25
db6	78.00	85.00	89.90	91.15
sym2	74.90	84.28	88.58	91.21
sym4	79.01	83.32	91.57	**93.37**
sym6	75.43	83.44	87.45	89.60
coif1	76.27	83.14	91.57	91.45
coif3	78.90	76.93	80.63	82.96
bior2.2	77.82	86.61	88.64	86.07
bior2.4	74.30	88.16	92.77	93.19

As shown in Figs. 3 and 4, with the increase of decomposition scale, the overall prediction accuracy obtained by experiments is improved under different conditions of wavelet basis functions. When the decomposition scales are 4 and 5, the overall prediction accuracy obtained by the experiment is notably close, which indicates that with the increase of the scale, the overall prediction accuracy will tend to be stable, will not continue to increase, or even may decline. Moreover, it can be seen from the Figs. 3 and 4 that although the choice of decomposition scale and wavelet basis function will affect the overall prediction accuracy of the experiment, the influence of the decomposition scale is greater than that of the wavelet basis function.

### Comparison with 1-D wavelet denoising

**Fig. 5:**
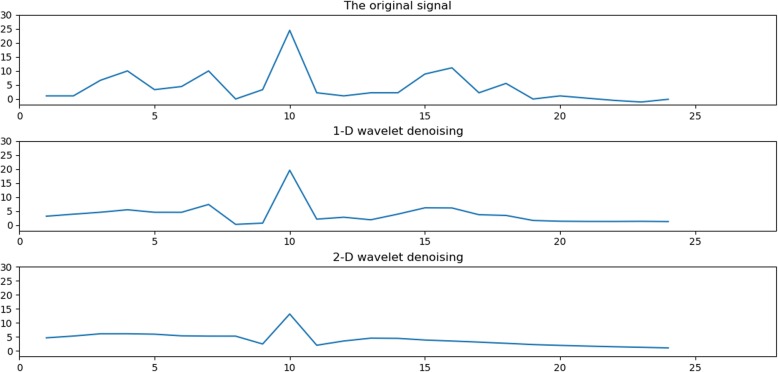
Comparisons of 1-D wavelet denoising and 2-D wavelet denoising on the 25PDB

To verify the superiority of the two-dimensional (2-D) wavelet denoising method, we compare it with the one-dimensional (1-D) wavelet denoising method. The 1A1W structural protein sequence in the 25PDB was selected as the experimental sample to compare the denoising effect. The decomposition scale is 5, and the sym4 wavelet is chosen as the wavelet basis function. The K value in the classifier KNN is still 3. We use the 24-dimensional numerical feature vectors extracted from the 1A1W protein sequence through the type 2 pseudo amino acid composition as the original signal. to intuitively show the comparison of the two denoising effects, we choose the form of graph to show. The comparison results of one-dimensional wavelet denoising and two-dimensional wavelet denoising are shown in Fig. 5.

As seen from Fig. 5, the original signal is notably messy, because it contains considerable redundant information, therefore, it seems to fluctuate. After 1-D wavelet denoising, although the signal has changed, the effect of denoising is not strong. After 2-D wavelet denoising, the signal is clearly different from the original signal, becoming smoother and more stable, indicating that the effect of denoising is notably good. This finding is observed in our study. We use variance to accurately describe the difference within the signal. The variance of the original signal is 30.526. After one-dimensional wavelet denoising, the variance of the signal is 14.274. After two-dimensional wavelet denoising, the variance of the signal becomes 6.189. In summary, the denoising effect of the 2-D wavelet is better than that of the 1-D wavelet.

To sum up, two-dimensional wavelet denoising is better than one-dimensional wavelet denoising, and this 2-D wavelet denoising method can be used not only in structural classes but also in other types of protein classification models.

### Selection of the K value in the K-nearest neighbor classifier

K- nearest neighbor classifier, which is based on the similarity of sample points to select the first K sample points for voting classification. However, this K value is often unknown, and choosing different K values will produce different prediction results. Therefore, to obtain better prediction results, it is necessary to select the optimal K value. In this section, the optimal K value is selected from 1 to 9. Under different K values, the prediction accuracy of each class and the overall prediction accuracy of the protein structure class sequence are shown in Table 7. Under different K values, the prediction accuracy of each class and the overall prediction accuracy of the protein structure class sequence are shown in Table 7.

**Table 7:** Prediction results by choosing different values of K on the 25PDB

Class	K								
	Jackknife test(%)								
	1	2	3	4	5	6	7	8	9
all-α	97.97	98.65	95.71	96.84	93.23	94.36	93.91	94.58	93.00
all-β	98.87	99.10	98.65	98.87	98.42	98.65	98.65	98.87	98.65
α/β	97.98	97.40	95.67	96.24	93.93	94.80	93.64	93.64	92.77
α + β	97.51	89.80	94.78	89.11	89.57	85.71	86.17	83.45	85.26

**Fig. 6:**
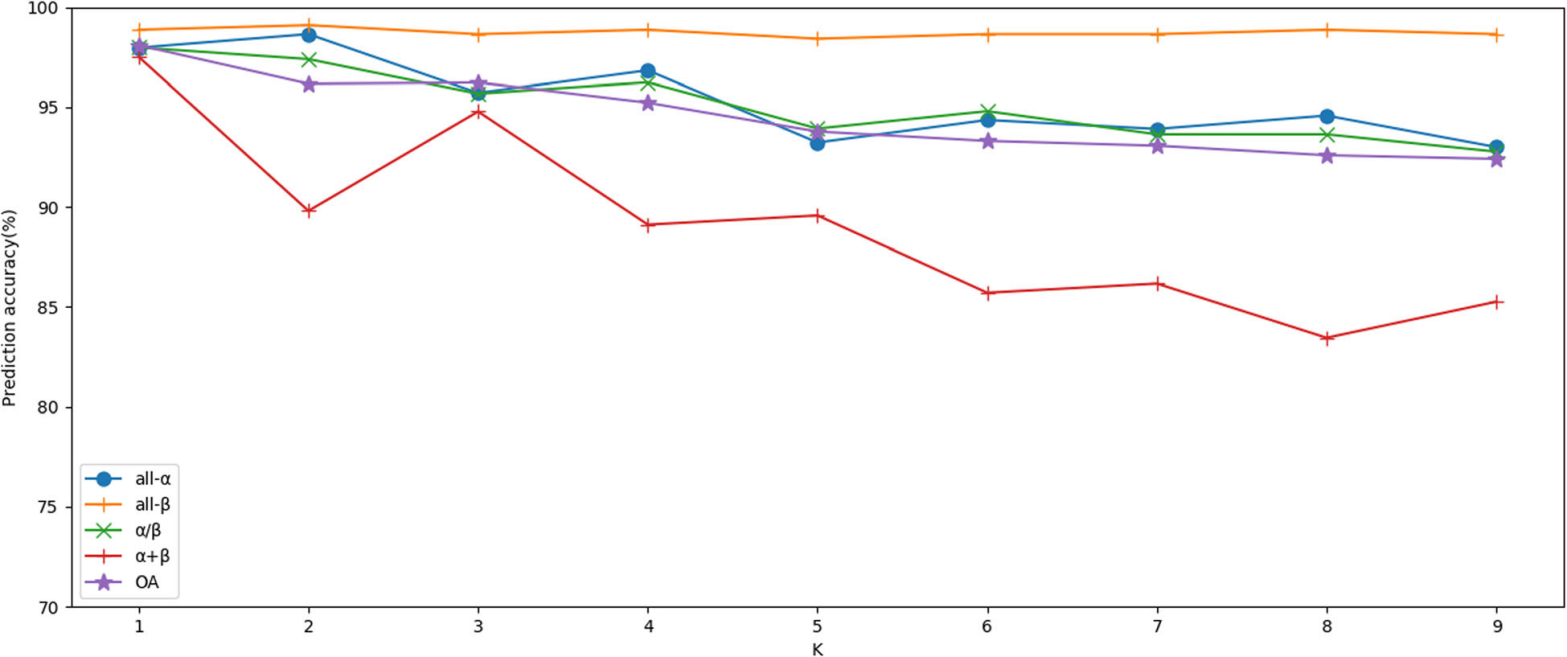
Prediction results by choosing different values of K on the 25PDB

As shown in Table 7, different K values have a certain impact on the prediction results. In model 1, with the increase of K values, the overall prediction accuracy decreases. When K is 1, the overall prediction accuracy is the highest, 97.91%, while when K is 9, the overall prediction accuracy is the lowest, 91.33%. To visualize the overall prediction accuracy under different K conditions, we use a line chart to describe it, as shown in Fig. 6. From the Fig. 6, it is clear that different K values will affect the prediction results of the experiment, and with the increase of K values, the overall prediction accuracy has a downward trend.

### Comparison of different strategies

In this paper, a feature fusion model based on parallel two-dimensional wavelet denoising is proposed. To better demonstrate the improvement of the prediction accuracy of the models, this section compares with other strategies.

**Fig. 7:**
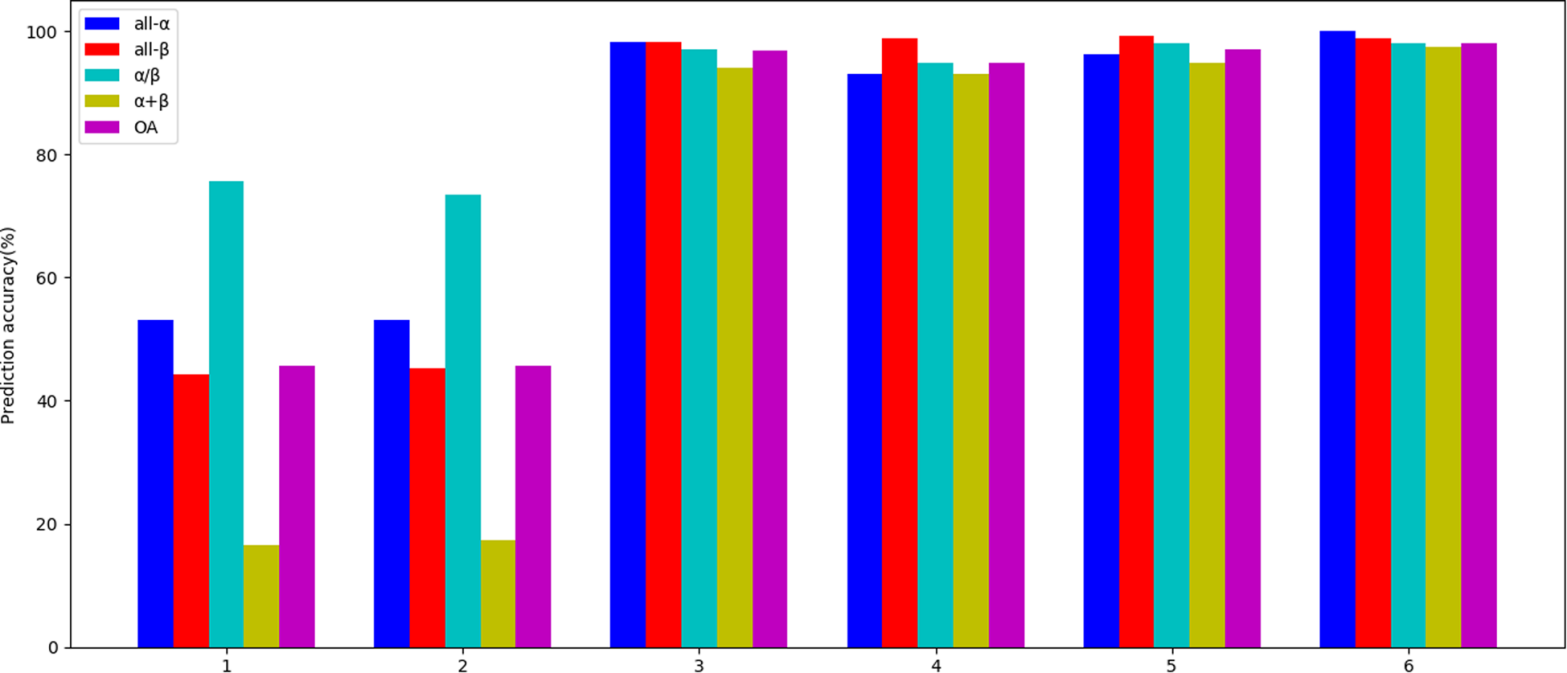
Comparison of different strategies on the 25PDB

Compare various strategies on the 25PDB. In the table, strategy 1 refers to the use of type 1 pseudo amino acid composition only; strategy 2 refers to the use of type 2 pseudo amino acid composition only; strategy 3 refers to the combination of type 1 pseudo amino acid composition and two-dimensional wavelet denoising; strategy 4 refers to the combination of type 2 pseudo amino acid composition with two-dimensional wavelet denoising; and strategy 5 refers to the first combination of features extracted from type 1 and type 2 pseudo amino acid composition. The feature vector fusion is then combined with two-dimensional wavelet denoising; strategy 6 refers to the model proposed in this paper. Among these strategies, the parameters λ and *r* in the two types of pseudo amino acid composition are both 2. In the classifier, the K value in KNN ranges from 1 to 9, and the parameters in two-dimensional wavelet denoising are also the best denoising wavelet basis function and decomposition scale. The experimental results are shown in Table 8 and Fig. 7.

**Table 8:** Comparison of different strategies on the 25PDB

Dataset	Prediction accuracy(%)					
	Strategy	all-α	all-β	α/β	α + β	OA
25PDB	1	53.05	44.24	75.72	16.55	45.79
	2	53.05	45.37	73.41	17.23	45.79
	3	98.19	98.19	97.11	94.10	96.89
	4	93.00	98.87	94.80	92.97	94.92
	5	96.16	99.32	97.98	94.78	97.01
	**6**	**99.97**	**98.87**	**97.98**	**97.51**	**98.09**

From Table 8 and Fig. 7, it can be seen that the overall prediction accuracy of model 1 proposed in this paper reaches the highest level, 98.09%, and it can be seen from the table that the idea of parallel two-dimensional wavelet denoising proposed in this chapter is effective. Compared with strategy 5, first fusing feature vectors and then denoising, the overall prediction accuracy is improved by 1.08%, while the application of two-dimensional wavelet denoising improves the prediction accuracy by 1.08%. The measurement results have a great impact. Strategy 1 and Strategy 2 do not use two-dimensional wavelet denoising, and their prediction accuracy is far from that of other strategies. In conclusion, the fusion idea proposed in this model is highly effective.

### The influence of different classifiers on prediction results

**Fig. 8:**
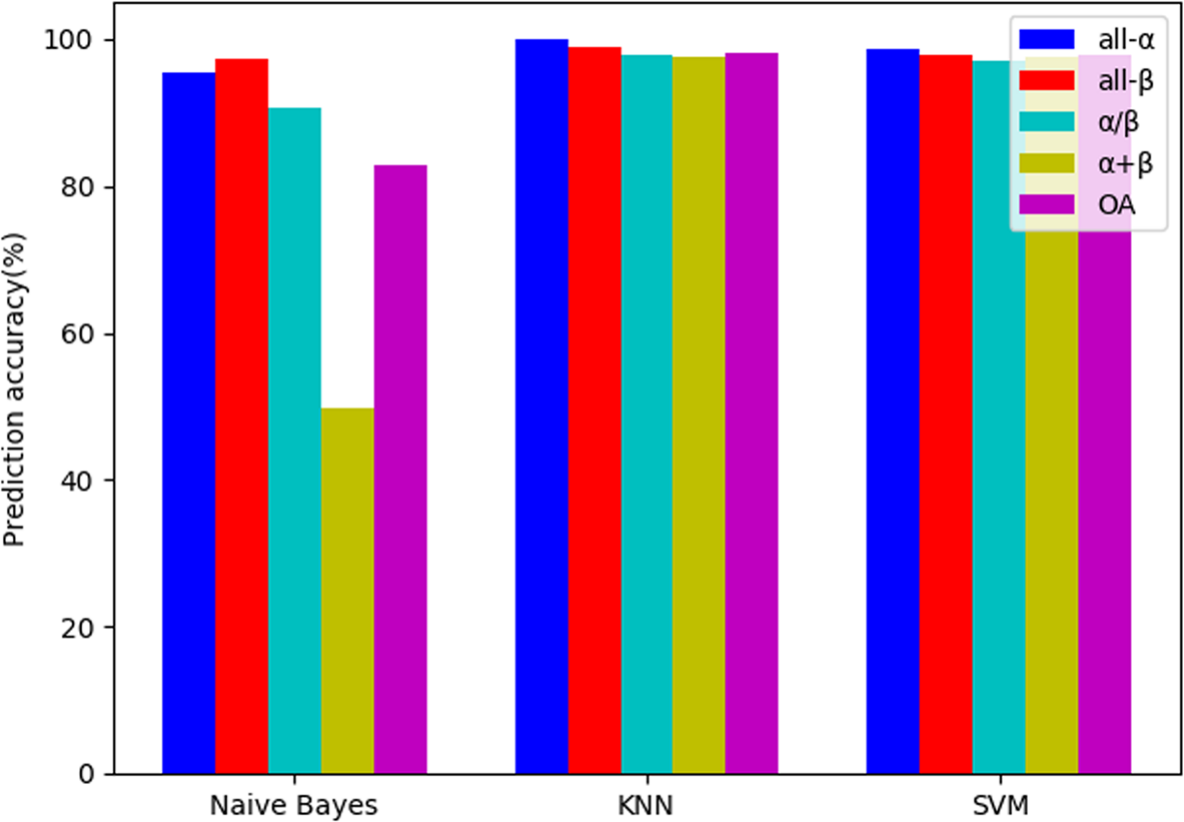
Influence of different classifiers on prediction results on the 25PDB

Three classifiers: Naive Bayes, KNN and SVM are used to explore the effects of different classifiers on the prediction results. The parameters of two types of pseudo amino acid composition are 2. The denoising parameters of two-dimensional wavelet denoising for the extracted feature vectors of type 1 pseudo amino acid composition: the wavelet basis function is db4 wavelet, the decomposition scale is 5, and the denoising parameters of two-dimensional wavelet denoising for the extracted feature vectors of type 2 pseudo amino acid composition: the wavelet basis function is sym4, and the decomposition scale is 5. The K value of KNN is the best 1. For SVM, the radial basis function is used as the kernel function, and the grid search strategy is used for the selection of C and G parameters. The search ranges of both are 2^− 10^ to 2^10^. The jackknife method was used to test the influence of three classifiers on the prediction results on the 25PDB. The experimental results are shown in Table 9 and Fig. 8.

**Table 9:** Influence of different classifiers on prediction results on the 25PDB

Classifier	Prediction accuracy(%)				
	all-α	all-β	α/β	α + β	OA
Naive Bayes	95.49	97.29	90.75	49.66	82.90
KNN	**99.97**	**98.87**	**97.98**	**97.51**	**98.09**
SVM	98.65	97.97	97.11	97.51	97.85

As shown in Table 9 and Fig. 8, when the KNN is used as the classifier, the overall prediction accuracy is the highest, 98.09%. The prediction accuracy of each category is the highest, and only the prediction accuracy of the α + β class is the highest in parallel with other categories. When Naive Bayes is used as the classifier, the overall prediction accuracy is 82.90%, which is considerably less than the KNN. This finding shows that the Naive Bayes is not as effective as the KNN in this experimental condition. When SVM is used as the classifier, the overall prediction accuracy is 97.85%. The possible reason for this finding is that the range of the parameter search is not appropriate, which causes the performance of SVM not to be as good as that of KNN. Moreover, SVM takes considerably more time to find parameters than KNN; therefore, considering the classifiers comprehensively, the classifier of this model chooses KNN.

### Prediction performance of our method

The performance of a method determines whether it can be applied by everyone. Therefore, as our study is no exception, the traditional performance evaluation methods are utilized to verify the performance of our methods. In model 1, based on two types of pseudo amino acid composition methods and parallel 2-D wavelet denoising, a machine learning prediction model with the fusion of two features is proposed, which is called PWD-FU-PseAAC. First, the feature information of protein sequences is extracted by type 1 pseudo amino acid composition and type 2 pseudo amino acid composition; in other words, the primary protein sequences are converted into 20 + λ dimensional and 20 + 2*r* dimensional numerical vectors respectively. Second, the 2-D wavelet denoising method is used to denoise the two feature vectors separately and remove their redundancy. Then, the two feature vectors after denoising are fused, which refers to splicing the first and last vectors of the two parts to form 40 + λ + 2*r* dimensional feature vectors. Finally, the optimal feature vectors are input into the KNN classifier for prediction, and the results are verified by jackknife. The optimal parameters of the prediction model can be obtained from the previous experimental analysis. The values of λ and *r* in both types of PseAAC are 2. The db4 wavelet is used as the wavelet function, and 5 is chosen as the decomposition scale to denoise the feature vectors extracted by type 1 PseAAC; Sym4 is chosen as the wavelet function and 5 is chosen as the decomposition scale to denoise the feature vectors extracted by type 2 PseAAC. The K value in the classifier is set to 1. The performance of the model is evaluated on the 25PDB, the 1189PDB and the 640PDB. The experimental results are shown in Table 10.

**Table 10:** Prediction performance of model 1 on three benchmark datasets

Dataset	Class	Sens(%)	Spec(%)	MCC	OA(%)
25PDB	all-α	97.97	99.84	0.983	98.09
	all-β	98.87	99.84	0.989	
	α/β	97.98	99.17	0.967	
	α + β	97.51	98.62	0.957	
1189	all-α	98.21	99.66	0.980	97.25
	all-β	99.32	99.87	0.993	
	α/β	99.10	97.23	0.950	
	α + β	91.29	99.41	0.930	
640	all-α	95.65	99.20	0.954	96.09
	all-β	98.05	99.59	0.979	
	α/β	97.18	96.98	0.928	
	α + β	93.57	98.93	0.936	

The results of four standard performance measures are shown in Table 10. From the results that emerged in Table 10, we note that we acquire 98.09, 97.25 and 96.09% overall accuracy on the 25PDB, the 1189PDB and the 640PDB, respectively. The overall accuracy obtained on three datasets was highly satisfactory. Moreover, the Matthews correlation coefficient (MCC) of α + β class proteins are lower than other classes for the three datasets. Hence, there are many challenges to identifying protein sequences of the α + β class with high very efficacy.

### Comparison with existing methods

To objectively compare our method with previously reported methods, we carried out experiments under the same conditions as the previous methods. Among these methods, the MEDP [58] method is based on evolutionary information, and a new feature expression method is proposed. The SCPRED [22] method is based on predictive secondary structure to construct new feature vectors. The PKS-PPSC [59] method is based on predictive secondary structure to construct feature vectors, but it uses chaotic game representation and information entropy to construct feature vectors. The method reported by Zhang et al. [23] is based on predictive secondary structure information, based on this information, the TPM matrix feature representation is proposed. The PSSS-PSSM [25] method combines predicted secondary structure features with the PSSM matrix. The PSSS-PsePSSM [60] method combines predicted secondary structure features with improved PSSM matrix, and proposes a new fusion feature expression. The WD-PseAAC [53] method extracts feature vectors based on SVM, using a single feature expression method and then denoises them with wavelet denoising. Our method is to denoise the extracted feature vectors and then fuse them.

**Fig. 9:**
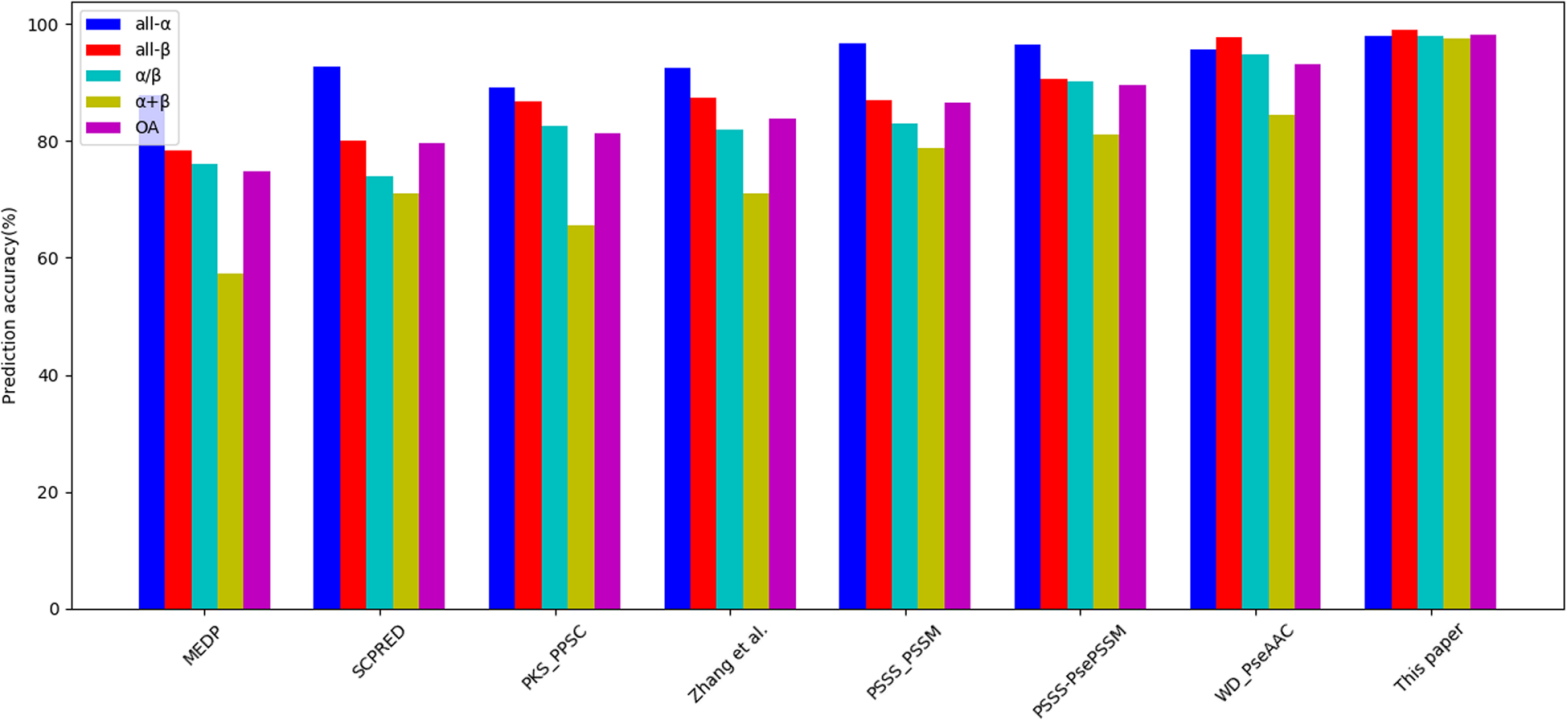
Comparison with other methods on the 25PDB

**Fig. 10:**
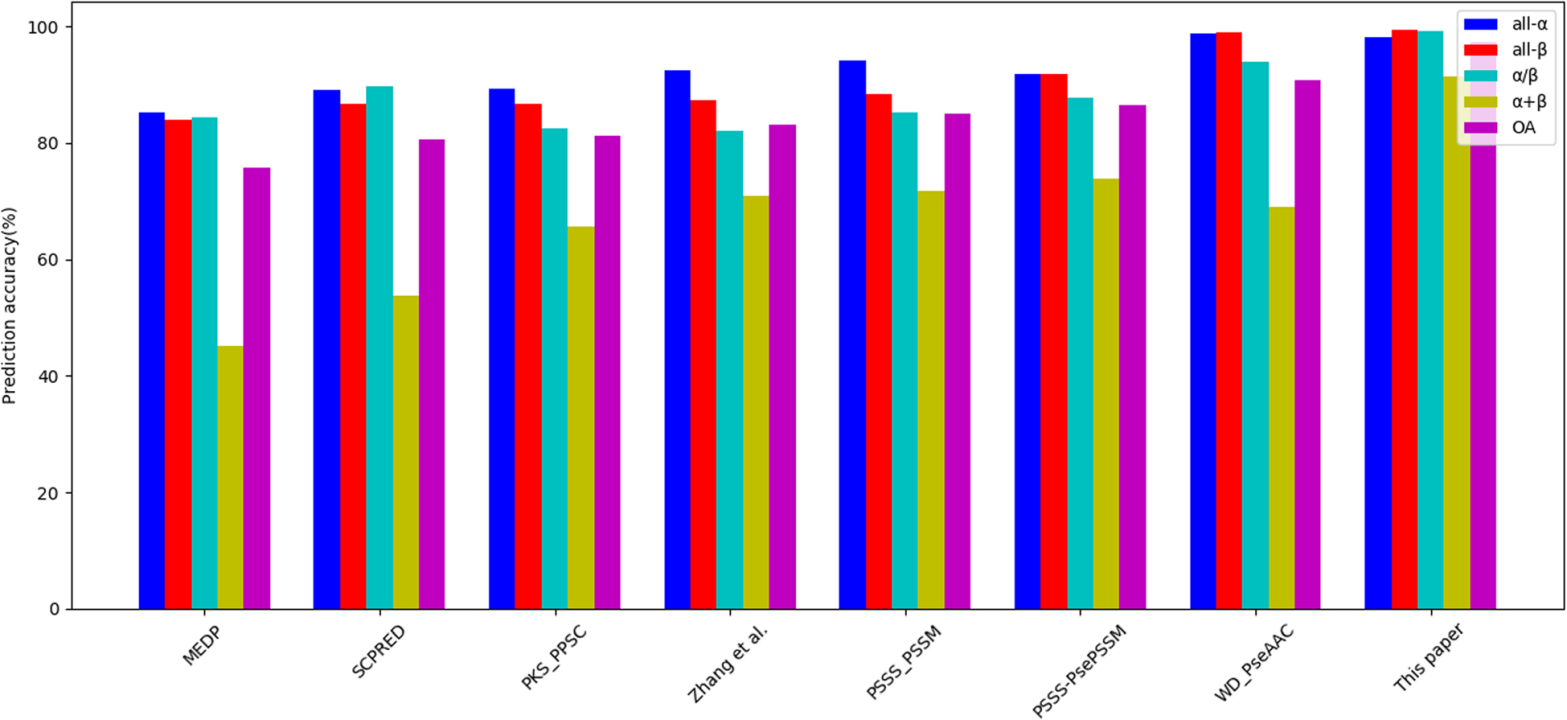
Comparison with other methods on the 1189PDB

**Fig. 11:**
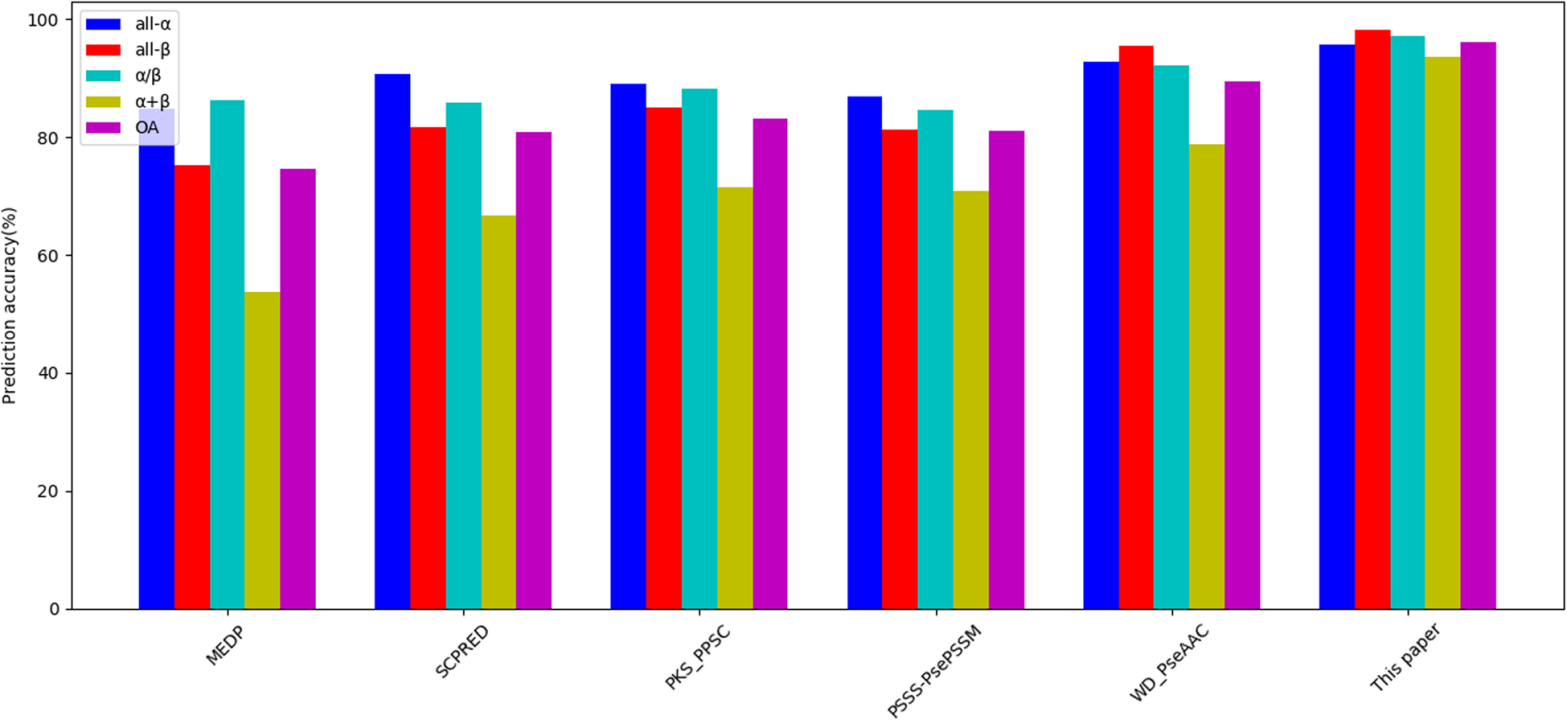
Comparison with other methods on the 640PDB

The experimental results are summarized in Table 11 and Figs. 9, 10, 11. From the experimental results in Table 11 and Fig. 9, the overall prediction accuracy of 98.1% is gained on the 25PDB, which is the highest and 5.0 to 23.3% higher than those of other methods. Furthermore, from the experimental results in Table 11 and Fig. 10, the overall prediction accuracy of 97.3% is also obtained on the 1189PDB, which is the highest and 6.5 to 21.5% higher than those of other methods. Moreover, from the experimental results in Table 11 and Fig. 11, the prediction results are also satisfactory for the 640PDB. The prediction accuracy of the four classes is the highest, and the overall prediction accuracy is the highest, 95.0%. At the same time, there are other significant changes that deserve our attention. For example, the overall prediction accuracy of our method can achieve such good results on three datasets because we have greatly enhanced the prediction rates of α/β class proteins and α + β class proteins, while the prediction rates of other methods for α/β class proteins and α + β class proteins are notably low. One of the reasons that the overall prediction accuracy of protein structural classes has been relatively low is that it is difficult to predict α/β and α + β proteins.

**Table 11:** Comparison with other methods on three benchmark datasets

Dataset	Prediction accuracy(%)					
	Method	all-α	all-β	α/β	α + β	OA
25PDB	MEDP [58]	87.8	78.3	76.0	57.4	74.8
	SCPRED [22]	92.6	80.1	74.0	71.0	79.7
	PKS-PPSC [59]	89.2	86.7	82.6	65.6	81.3
	Zhang et al. [23]	92.4	87.4	82.0	71.0	83.9
	PSSS-PSSM [25]	96.6	87.1	83.0	78.9	86.6
	PSSS-PsePSSM [60]	96.4	90.5	90.2	81.2	89.5
	WD-PseAAC [53]	95.7	97.7	94.8	84.4	93.1
	**This paper**	**98.0**	**98.9**	**98.0**	**97.5**	**98.1**
1189	MEDP [58]	85.2	84.0	84.4	45.2	75.8
	SCPRED [22]	89.1	86.7	89.6	53.8	80.6
	PKS-PPSC [59]	89.2	86.7	82.6	65.6	81.3
	Zhang et al. [23]	92.4	87.4	82.0	71.0	83.2
	PSSS-PSSM [25]	94.2	88.4	85.3	71.8	85.0
	PSSS-PsePSSM [60]	91.9	91.8	87.7	73.9	86.6
	WD-PseAAC [53]	98.7	99.0	94.0	68.9	90.8
	**This paper**	**98.2**	**99.3**	**99.1**	**91.3**	**97.3**
640	MEDP [58]	84.8	75.3	86.4	53.8	74.7
	SCPRED [22]	90.6	81.8	85.9	66.7	80.8
	PKS-PPSC [59]	89.1	85.1	88.1	71.4	83.1
	Zhang et al. [23]	–	–	–	–	–
	PSSS-PSSM [25]	–	–	–	–	–
	PSSS-PsePSSM [60]	87.0	81.2	84.7	70.8	81.0
	WD-PseAAC [53]	92.8	95.5	92.1	78.9	89.5
	**This paper**	**95.7**	**98.1**	**97.2**	**93.6**	**96.1**

In summary, through the analysis of the above experimental results, we can conclude that our models can efficaciously forecast the structural classes of protein sequences, even on the low-similarity datasets. The reason why our method is better than others is that although the traditional method is used to extract feature vectors, the feature extraction method that we adopt may not be as good as others. However, after feature extraction, we use two-dimensional wavelet denoising to denoise the redundant information in the feature vector, which makes it more recognizable. In addition, other researchers also use the method of wavelet denoising, but this paper proposes a new fusion strategy based on wavelet denoising.

## Conclusions

A new method, PWD-FU-PseAAC, is proposed to forecast the structural classes of protein sequences. The method ameliorates the shortcomings of traditional feature expression methods, which contain considerable redundant information that cannot result in inefficiency. Therefore, in this paper, a new idea of fusion has been proposed, in which a parallel 2-D wavelet denoising algorithm is adopted to process the extracted feature vectors before fusing them. Through related experiments, we not only verify the effect of the wavelet denoising algorithm on the models but also compare the overall accuracy of our models with those of other methods. Ultimately, we can conclude that our method is good for predicting the structural classes of protein sequences and is expected to be applied in other fields related to bioinformatics [61–74]. The related source codes and datesets are available at https://github.com/Xiaoheng-Wang12/Wang-xiaoheng/tree/master.

## Data Availability

The related source codes and datasets are available at https://github.com/Xiaoheng-Wang12/Wang-xiaoheng/tree/master.

## Abbreviations

1-D: One dimentonal
2-D: Two dimentonal
ACC: Amino acid composition
KNN: K-nearest neighbors
MCC: Matthews correlation coefficient
OA: Overall accuracy
PseACC: Pseudo amino acid composition
Sens: Sensitivity
Spec: Specificity
SVM: Support vector machines
